# Fuzzy topological analysis of fuzzy Helm graphs with applications to protein interaction networks

**DOI:** 10.1038/s41598-026-61816-9

**Published:** 2026-07-15

**Authors:** Zeeshan Saleem Mufti, Ali H. Tedjani, Shama Liaqat, Gamachu Adugna Ganati

**Affiliations:** 1 Department of Mathematics and Statistics, The University of Lahore, Lahore, Pakistan; 2 Department of Mathematics and Statistics, College of Science, Imam Mohammad Ibn Saud Islamic University (IMSIU), Riyadh, 11564 Saudi Arabia; 3 Department of Mathematics, Wallaga University, Nekemte, Ethiopia

**Keywords:** Fuzzy graph, Fuzzy topological index, Helm graph, Fuzzy Helm graph, Computational biology and bioinformatics, Engineering, Mathematics and computing

## Abstract

In biological and chemical networks, in which the strength of interactions is often not absolute, uncertainty is an inherent feature of many real-world networks. A fuzzy graph consists of vertices with fuzzy memberships and fuzzy relationships among vertices and edges; therefore, fuzzy graph theory is a natural approach for modelling systems in which the connections between the vertices are fuzzy or not precisely known. Helm graphs are constructed by adding pendant vertices to the outer cycle of a wheel graph and have a layered hierarchy similar to that found in hub-and-spoke networks, such as those of protein interaction and communication networks. Encouraged by this structural similarity, in the present study, some degree-based fuzzy topological indices are investigated on fuzzy Helms graphs. Specifically, we obtain closed-form analytical expressions for the fuzzy Zagreb, fuzzy Randić, fuzzy Harmonic, fuzzy *F*-index, fuzzy Sombor, fuzzy Misbalance Prodeg, and fuzzy Nirmala indices. The formulas are derived directly from the partition of the type of vertices of the fuzzy Helm graph and are checked symbolically and by a numerical example. The deduced expressions show the dependence of these descriptors on structural properties, such as pendant attachments, edge weight distribution, and connectivity of the hubs. As a concrete application, the proposed framework is used in the case of the P53 protein interaction network, a central pathway in cancer biology. The numerical results illustrate the ability of fuzzy descriptors to differentiate between the dominant regulatory role of P53 and the role played by interacting partners, which cannot be achieved by classical crisp indices because they lose the graded aspect of biological interaction confidence. Therefore, this study suggests the use of fuzzy topological indices for network analysis in a wider context and suggests the natural development of the said approach to other families of fuzzy graphs, as well as to uncertain networks in chemical and biological applications.

## Introduction

Graph theory is a prominent mathematical tool used for modeling the relationships and interactions between objects in science, engineering, computer science, chemistry, and biological systems. While many real-world networks represent the relationships between entities in a graph structure, such a structure is an efficient representation of pairwise relationships. However, many networks in the real world are uncertain, vague, and partially connected, and cannot be represented by a crisp graph structure. To overcome these deficiencies, fuzzy graph theory fuses graph theory with fuzzy set theory, whereby unclear relationships are represented by membership values associated with vertices and edges. In 1965, Zadeh^7^ presented the concept of fuzzy sets, which offers a mathematical representation of imprecise and uncertain information. In 1975, Rosenfeld generalized this concept to fuzzy graphs^9^ where vertices and edges have degrees of membership rather than a binary relation. Since then, fuzzy graph theory has become an active research area owing to its applicability in communication systems, social networks, transportation systems, medical diagnosis, and biological interaction networks.

Recently, researchers have been interested in generalizing classical topological indices to fuzzy environments for the analysis of uncertain structures. Some degree-based fuzzy topological indices, such as fuzzy Zagreb indices, fuzzy Randić index, fuzzy Harmonic index, fuzzy Sombor index, and fuzzy F-index, have been used successfully in chemistry, decision making, and network analysis. These descriptors offer more detailed information on the structure because they explicitly account for uncertainty in the graph structure. Moreover, fuzzy graph theory has been used extensively in recent years to model generalized fuzzy graph structures, which are beneficial for the representation of complex systems with uncertain interactions and incomplete information^18,19^.

Helm graphs have highly symmetric configurations and rich topological properties and are appropriate for the study of structural behavior under fuzziness among various graph structures. A Helm graph is obtained by attaching pendant vertices to every outer cycle vertex of a wheel graph Wn, k. Although Helm graphs have been investigated in classical graph theory, their fuzzy counterparts remain insufficiently explored, particularly in fuzzy topological descriptors. Comprehensive studies on fuzzy Helm graphs are still limited, and the existing literature is mainly dedicated to fuzzy flower, pizza, and qCn graphs, and related structures.

Motivated by these observations, this study presents a detailed investigation of several fuzzy topological indices for the fuzzy Helm graph. Closed-form expressions for the considered indices were derived and analyzed theoretically. In addition, a practical application involving the P53 protein interaction network is presented to demonstrate the effectiveness of fuzzy Helm graphs in modeling uncertain biological systems.

One of the most important proteins encoded by TP53 is the tumor suppressor protein P53. It is a well-studied protein in the field of molecular biology. The Kruskal algorithm for computing a minimum spanning tree of a weighted graph was influential^6^ and Prim later developed another efficient algorithm for computing a minimum spanning tree of a weighted graph^5^. Zadeh presented a theory of fuzzy sets $\mathcal {A}$ on a universe of discourse $\mathcal {X}$ which offers a mathematical tool for dealing with uncertainty and vagueness^8^. Compared to the classical set, in a fuzzy set, each element $x\in \mathcal {X}$ has a membership degree $\mu _{\mathcal {A}}(x)\in [0,1]$. This formulation makes partial membership possible and allows for more realistic modelling of imprecise phenomena in the real world. Thus, this membership function is a vital component in defining the “membership” of an element in a fuzzy set.

To extend the concept of fuzzy sets, fuzzy relations were introduced to represent uncertain relationships between elements. A fuzzy relation between two sets $\mathcal {X}$ and $\mathcal {Y}$ is defined by the membership function

$$\mu _{\mathcal {R}}:\mathcal {X}\times \mathcal {Y}\rightarrow [0,1],$$

is a measure of the association between every ordered pair *(x,y)* in $\mathcal {X}\times \mathcal {Y}$. It is a generalization of the classical binary relation, where the degrees of connectivity vary. Topological indices are numerical descriptors of molecular graphs that have been extensively used to characterize molecular and chemical properties. One of the earliest and most influential contributions to chemical graph theory was made by Wiener in 1947 through the introduction of the Wiener index for predicting the boiling points of paraffin compounds^10^. Later, Gutman and Trinajstić employed graph-theoretical methods to calculate the total number of *π*-electrons in alternant hydrocarbons using the adjacency matrix of the molecular graph^11^.. In 1975, Milan Randić introduced the Randić index, also known as the connectivity index, as a measure of the branching nature of molecular structures^12^. This index exhibits strong correlations with several physicochemical properties, including molecular stability and reactivity, and is defined using the reciprocal square root of the degrees of adjacent vertices. Then, several degree-based topological indices were proposed and analyzed. Estrada studied the characterization of three-dimensional molecular structures (3-D)^16^ and Dobrynin studied the structural properties of hexagonal systems using the Wiener index (3-D)^15^. Mondal and Pal investigated graphene structures using graph-theoretical methods and proposed new topological descriptors based on degree measures to study the structural properties of graphene^13^. They also designed and calculated other descriptors in another study, which provided more insight into the molecular topology of the studied molecules^14^. Amin and Nayeem studied the F-index and F-coindex of line graphs of subdivision graphs^17^. Idrees *et al.* illustrated the importance of topological indices in the drug discovery process for eye diseases^33^ and Hasani explored the correlation between topological indices and quantitative structure property relationships (QSPR) of the compounds used in the treatment of heart diseases^34^. Many topological descriptors are already known in classical graph theory; however, they are not general enough to accurately describe the uncertainty in relationships. As fuzzy logic has become increasingly integral to various scientific applications, it is natural to extend classical topological concepts to fuzzy graph settings. In 2020, Kalathian studied a new family of fuzzy topological indices, which are extensions of the degree-based indices for fuzzy graphs^18^. They introduced vertex and edge membership values into the computation of graph descriptors to provide a more complete analysis of uncertain graph structures. Later, Atef and Nawar studied the relationship between fuzzy graphs and fuzzy topological spaces and how fuzzy graphs can be used to define and analyze fuzzy topological indices^19^. Their work enhanced the theory and utilization of fuzzy graph theory.

In recent years, there has been tremendous progress in fuzzy graph theory, which has increased the range of applications of fuzzy topological indices for complex and uncertain systems. Several generalized fuzzy graph structures have been studied, and new descriptors have been proposed to study their structural behavior under uncertainty. Fuzzy neighborhood degree-based indices, fuzzy distance-based descriptors, and hybrid fuzzy graph models have been studied and shown to be useful in chemistry, communication systems, and biological modelling. Additionally, classical topological descriptors have been extended to fuzzy situations, as they can be more informative about the uncertainty in real-world interactions. These developments spur the study of fuzzy graph families that contain both symmetry and uncertainty, such as fuzzy Helm graphs, which would be of interest for further mathematical characterizations of such families, as suggested in^38–40^.

Islam and Pal introduced another study, where they introduced the hyper-Wiener index, a new index for fuzzy graphs, and discussed its various applications, especially in the share market^20^. The authors presented the results of calculations using various hydrocarbon structures to demonstrate the applicability of the fuzzy Zagreb indices in molecular topology. Ahmad et al.^21^ used Fuzzy Randić and Fuzzy Harmonic indices to solve Cyber Crime problems, showcasing the use of these indices to detect patterns, relationships and vulnerabilities of Cyber networks.

Subsequently, Tabraiz et al.^22^ investigated and calculated some fuzzy topological indices specifically for the fuzzy flower graph. These indices represent various structural properties of a fuzzy flower graph, providing insights into the connectivity and characteristics of the graph. Islam and Pal proposed a detailed mathematical formula for calculating the F-index in fuzzy graphs, including the effect of membership values of nodes and edges on the computation of the F-index^23^. Mufti et al.^24^ calculated some fuzzy topological indices of $qC_n$ graph in 2023, providing an understanding of structural properties such as connectivity and branching. Ahmad developed a methodology for multi criteria decision making based on the fuzzy indices defined, which can be used to assess and prioritize alternatives, taking into consideration the uncertainty in the data^25^.

Mufti presented a thorough mathematical analysis of fuzzy indices, exploring their properties and relationships within the context of a pizza graph^26^. In, the authors provided a detailed mathematical formulation for calculating the second Zagreb index in fuzzy graphs, along with relevant properties and theorems^27^. Kosari proposed a methodological framework that integrate fuzzy topological indices such as connectivity index, average connectivity index and Randić index for fuzzy graph^28^. Sarala found to reveal insight into the topological properties of fuzzy graphs by examining numerous topological indices^29^.

Jamil introduced an innovative application of intuitionistic fuzzy graph theory to enhance the efficiency of vaccination centers through fuzzy topological indices^35^. The Gutman index, based on degree and distance, was studied for fuzzy graphs^36^. Uzma et al.^37^ introduced Wiener index-based energies for fuzzy graphs using fuzzy wiener matrices and their eigenvalues. Bounds are established, and the approach is then applied to a multi-criteria decision-making problem. Mirza et al.^30^ introduced polynomial regression, a machine learning technique, to create polynomials for adjacency and degree matrices in the fuzzy conjugate graph of dihedral group, and simplifying the calculations. Recently, Liaqat et al.^32^ introduced the fuzzy misbalance prodeg index, an abstracted variant of the traditional misbalance prodeg index used in crisp graph theory. Compared to classical degree-based topological indices, fuzzy-based topological indices provide a more flexible and realistic framework for analyzing systems involving uncertainty, imprecision, and partial relationships. Classical graph descriptors generally assume that vertices and edges are either completely present or absent, which may not accurately represent many real-world networks, such as biological systems, communication networks, and chemical interaction structures. In contrast, fuzzy topological indices assign membership values to vertices and edges, allowing for a more effective quantification of the strength and reliability of interactions. This capability allows fuzzy indices to model imperceptible fluctuations in connectivity, uncertainty in interactions, and irregularities in the structure that crisp graph models cannot adequately represent. In addition, fuzzy descriptors provide better sensitivity in the identification of influential nodes, weak interactions, and hidden structural patterns; hence, they are highly applicable in the fields of chemistry, bioinformatics, medical diagnosis, and uncertain network analysis. This study establishes bounds for the given fuzzy index and analyzes specific classes of graphs. In the intuitionistic fuzzy graph framework, Imran introduced two types of Sombor indices with their properties, which would facilitate the application of these indices in practice^31^. The main reason for the present study being based on fuzzy-based topological indices is that some of the graph structures under consideration have uncertainties and varying interaction strengths that cannot be captured by simple (crisp) graph structures. Many real-world systems may be more complex than the relationships assumed by classical topological indices, which typically rely on the assumption of exact adjacency relations and fixed connectivity of vertices. Fuzzy topological indices, on the other hand, include membership values for vertices and edges, so that more realistic descriptions of uncertain and partially connected structures can be provided. The non-binary interactions found in all types of biological interaction networks, molecular systems, and communication models require the use of fuzzy graph descriptors to ensure added analytical power and better interpret the structure. In recent years, graph-theoretic descriptors and network-based analytical techniques have gained significant importance in the study of complex systems, molecular modeling, and computational network analysis^41,42^. The emerging application of topological and structural graph descriptors in uncertain and large-scale interaction networks is exemplified. In this study, the gap is filled by the analysis of the fuzzy Helm graph based on a complete list of fuzzy topological indices, namely fuzzy Zagreb, Randić, harmonic, F-index, misbalance prodeg, Sombor, and Nirmala indices. General formulas were obtained for each index and verified using specific examples. In addition, we suggest a new approach to the modelling of the ${\textbf {P}}53$ protein interaction network by the fuzzy Helm graph, which considers biological uncertainty. Several studies have explored the fuzzy topological descriptors of various families of graphs; however, relatively few have focused on fuzzy Helm graph structures. This study further extends the existing framework of degree-based fuzzy topological analysis to fuzzy Helm graphs and presents closed-form expressions for several descriptors in a unified framework, while showcasing their applications in a model of a biological network with uncertainty. Our work not only provides new theoretical contributions in the form of closed-form index formulas but also alleviates the gap between theory and application by providing examples of the use of fuzzy Helm graphs in biomedical data modeling. This positions our research as a meaningful extension of the state-of-the-art in both fuzzy graph theory and computational biology.

**Fig. 1:**
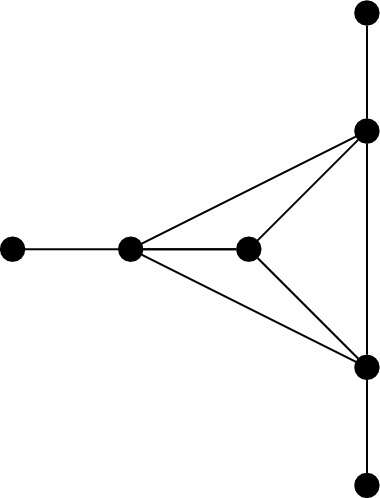
Helm Graph.

1. In *graph* $G_c$ (Crisp Graph) the structure is specified by dual values $G_c = (\alpha , \beta )$ consisting of:
  *α*, a cluster of nodes,
  *β*, set of links, that connects distinct pair of nodes.
  A graph is composed of nodes as its basic elements, whereas edges represent the connection among them.
2. Within graph theory, the degree of a node in $G_c$ corresponds to the number of edges $p_\alpha$ adjoining that node. The notion $\deg (p_\alpha )$ denotes the degree of node $p_\alpha$. The edge count at a node $p_\alpha \in \alpha$ in a graph $G_c = (\alpha , \mathcal {P_\alpha })$ is formally defined as
  $$\deg (p_\alpha ) = |\{ p_\alpha \in \mathcal {P_\alpha } \}|$$
  In this context, *| ~~~ |* signifies the size of the set.
3. We define the *Helm graph* $\mathcal {H}_{T_\alpha }$ as the structure produced by taking $T_\alpha$ wheel graph, and adding a pendant(leaf) vertex to all nodes on the outer rim of the wheel graph. See Fig. 1
4. A *fuzzy graph* $G_f$ can be seen as broader version of classical graph, where each node along with edge is described through membership degrees instead of absolute binary assignments. A fuzzy graph is a collection of $G_f = (\alpha _f, \beta _f)$ in which:
  A fuzzy collection of nodes $\alpha _f$ specified by association function $\mu _{\alpha _f}: \alpha \rightarrow [0, 1]$.
  The edge set in fuzzy graph, denoted by $\beta _f$ is specified through a mapping $\mu _{\beta _f}: \alpha \times \alpha \rightarrow [0, 1]$, indicating every edge possesses a membership value ranging from 0 to 1.
  The mapping $\mu _{\alpha _f}(p_\alpha )$ allocates a membership value to every node $p_\alpha \in \alpha _f$, which quantifies its degree of participation in the fuzzy structure. Similarly, the membership function $\mu _{\beta _f}(p_\alpha , q_\alpha )$ provides a membership value for every edge $(p_\alpha , q_\alpha ) \in \alpha _f \times \alpha _f$, quantifying the extent to which the pair $(p_\alpha , q_\alpha )$ participates as an edge in the fuzzy structure.
5. The concept of *order* in the fuzzy graph $G_f$ refers to the cardinality of its nodes collection $\alpha _f$, expressed as $|\alpha _f|$. Owing to the fuzziness of nodes, the *order* of a fuzzy graph may also be interpreted as the sum of the membership values of all nodes:
  $$|\alpha _f| = \sum _{p_\alpha \in \alpha _f} \mu _{\alpha _f}(p_\alpha )$$
6. For the fuzzy graph $G_f$, the *degree* of a node $p_\alpha$ is obtained by computing the aggregate membership values of all edges linked to $p_\alpha$:
  $$\deg (p_\alpha ) = \sum _{p_\alpha \in \alpha _f} \mu _{\beta _f}(p_\alpha , q_\alpha )$$
  This definition generalizes the classical notion of vertex degree by incorporating the fuzzy membership values of the nodes and the links between them.
7. For a fuzzy graph $G_f$, the *size* is given by the summation of membership values assigned to its edges:
  $$|\beta _f| = \sum _{(p_\alpha ,q_\alpha ) \in \alpha _f \times \alpha _f} \mu _{\beta _f}(p_\alpha , q_\alpha )$$
  It expresses the total connectivity in a fuzzy graph, reflecting the different strengths of its edges. The *Zagreb indices* are classical indices in chemical graph theory that describe the branching structure of molecules. To incorporate the inherent uncertainty of real-world systems, Kalathian introduced fuzzy versions of the Zagreb indices. For a fuzzy structure $G_f = (\alpha _f, \beta _f)$ these indices are formulated using mapping functions of edges along with nodes.
8. In the fuzzy graph, the *first fuzzy Zagreb index* $\mathbb {M}_1(G_f)$ is given by sum of node membership values multiplied by the squares of their degrees:
  $$\mathbb {M}_1(G_f) = \sum _{p_\alpha \in \alpha _f} \mu _{\alpha _f}(p_\alpha ) \cdot \left( \deg (p_\alpha ) \right) ^2$$
  In this expression, $\deg (p_\alpha )$ refers to the degree of the node $p_\alpha$ in $G_f$, and $\mu _{\alpha _f}(p_\alpha )$ indicates its membership grade. The fuzzy degree of each vertex is computed using the membership values of the incident edges. Therefore, edge membership information is implicitly incorporated through the fuzzy degree in the following descriptors.
9. A *second fuzzy Zagreb index* $\mathbb {M}_2(G_f)$ is computed as half the sum of the products of linked node degree along with membership values, reflecting the fuzzy nature of the structure:
  $$\mathbb {M}_2(G_f)=\frac{1}{2} \sum _{(p_\alpha ,q_\alpha ) \in \beta _f} \left( \mu _{\alpha _f}(p_\alpha )\cdot \deg (p_\alpha ) \right) \left( \mu _{\alpha _f}(q_\alpha )\cdot \deg (q_\alpha ) \right) ,$$
  Here, $p_\alpha$ and $q_\alpha$ possess degrees $\deg (p_\alpha )$ and $\deg (q_\alpha )$, and their membership grades are $\mu _{\alpha _f}(p_\alpha )$ and $\mu _{\alpha _f}(q_\alpha )$. In this study, the fuzzy edge set $\beta _f$ is treated as a symmetric fuzzy relation on the ordered vertex pairs. Consequently, each undirected edge is counted twice in the corresponding summation. Therefore, the factor $\frac{1}{2}$ is included in several edge-based fuzzy topological indices to avoid double-counting.
10. Given a fuzzy graph $G_f = (\alpha _f, \beta _f)$ and its *fuzzy Randić index* $\mathbb {R}(G_f)$ is defined as the weighted sum:
  $$\mathbb {R}(G_f) = \frac{1}{2} \sum _{(p_\alpha ,q_\alpha ) \in \beta _f} \frac{1}{\sqrt{\mu _{\alpha _f}(p_\alpha ) \cdot \deg (p_\alpha ) \cdot \mu _{\alpha _f}(q_\alpha ) \cdot \deg (q_\alpha ) }}$$
11. considering the fuzzy graph $G_f$, the *fuzzy Harmonic index* $\mathbb {H}(G_f)$ takes the form:
  $$\mathbb {H}(G_f) =\frac{1}{2} \sum _{(p_\alpha ,q_\alpha ) \in \beta _f} \frac{1}{ \mu _{\alpha _f}(p_\alpha )\cdot \deg (p_\alpha ) + \mu _{\alpha _f}(q_\alpha )\cdot \deg (q_\alpha )}$$
12. The *fuzzy F-index* adapts the classical F-index to fuzzy graphs by taking into account both node fuzziness and degree. The fuzzy F-index $\mathbb {F}(G_f)$ associated with the fuzzy graph $G_f = (\alpha _f, \beta _f)$ is expressed as follows:
  $$\mathbb {F}(G_f) = \sum _{p_\alpha \in \alpha _f} \left( \mu _{\alpha _f}(p_\alpha ) \cdot \deg (p_\alpha ) \right) ^3$$
13. The fuzzy Misbalance Prodeg index $\mathbb{M}\mathbb{P}(G_f)$ corresponding to a fuzzy graph $G_f = (\alpha _f, \beta _f)$ is expressed as:
  $$\mathbb{M}\mathbb{P}(G_f) = \sum _{(p_\alpha ,q_\alpha ) \in \beta _f} \left( \sqrt{\mu _{\alpha _f}(p_\alpha )\cdot \deg (p_\alpha ) }+ \sqrt{\mu _{\alpha _f}(q_\alpha )\cdot \deg (q_\alpha )} \right)$$
14. The fuzzy Sombor index $\mathbb {S}(G_f)$ associated with fuzzy graph $G_f = (\alpha _f, \beta _f)$ is given by:
  $$\mathbb {S}(G_f) = \sum _{(p_\alpha ,q_\alpha ) \in \beta _f} \sqrt{ \left( \mu _{\alpha _f}(p_\alpha ) \cdot \deg (p_\alpha )\right) ^2 + \left( \mu _{\alpha _f}(q_\alpha ) \cdot \deg (q_\alpha )\right) ^2 }$$
15. Used as topological measure, the *fuzzy Nirmala index* helps in evaluating the structure of fuzzy graphs. Given a fuzzy structure $G_f = (\alpha _f, \beta _f)$, the fuzzy Nirmala index $\mathbb {N}(G_f)$ is expressed as
  $$\mathbb {N}(G_f) = \sum _{(p_\alpha ,q_\alpha ) \in \beta _f} \sqrt{\mu _{\alpha _f}(p_\alpha )\cdot \deg (p_\alpha )+\mu _{\alpha _f}(q_\alpha )\cdot \deg (q_\alpha )}$$

Table 1 summarizes the core fuzzy topological descriptors investigated in this study. For each index, its mathematical formulation and corresponding interpretations are provided. This overview explains the meaning of each index in fuzzy graphs and their structural importance, especially in characterizing connectivity, imbalance, and degree-based properties in the fuzzy graph.

**Table 1:** Overview of Fuzzy Topological indices Used in This Study.

**Index**	**Formula**	**Meaning**
First Fuzzy Zagreb Index	$\sum _{p_\alpha \in \alpha _f} \mu _{\alpha _f}(p_\alpha ) \cdot \left( \deg (p_\alpha ) \right) ^2$	Measures degree contribution over edges with fuzziness
Second Fuzzy Zagreb Index	$\frac{1}{2} \sum _{(p_\alpha ,q_\alpha ) \in \beta _f} \left( \mu _{\alpha _f}(p_\alpha )\cdot \deg (p_\alpha ) \right) \left( \mu _{\alpha _f}(q_\alpha )\cdot \deg (q_\alpha ) \right)$	Reflects product of fuzzy degrees across edges
Fuzzy Randić Index	$\frac{1}{2} \sum _{(p_\alpha ,q_\alpha ) \in \beta _f} \frac{1}{\sqrt{\mu _{\alpha _f}(p_\alpha ) \cdot \deg (p_\alpha ) \cdot \mu _{\alpha _f}(q_\alpha ) \cdot \deg (q_\alpha ) }}$	Indicates branching; smaller values imply higher branching
Fuzzy Harmonic Index	$\frac{1}{2} \sum _{(p_\alpha ,q_\alpha ) \in \beta _f} \frac{1}{ \mu _{\alpha _f}(p_\alpha )\cdot \deg (p_\alpha ) + \mu _{\alpha _f}(q_\alpha )\cdot \deg (q_\alpha )}$	Reflects uniformity in vertex degrees
Fuzzy Misbalance Prodeg Index	$\sum _{(p_\alpha ,q_\alpha ) \in \beta _f} \left( \sqrt{\mu _{\alpha _f}(p_\alpha )\cdot \deg (p_\alpha ) }+ \sqrt{\mu _{\alpha _f}(q_\alpha )\cdot \deg (q_\alpha )} \right)$	Quantifies imbalance in vertex degrees over fuzzy edges
Fuzzy F-index	$\sum _{p_\alpha \in \alpha _f} \left( \mu _{\alpha _f}(p_\alpha ) \cdot \deg (p_\alpha ) \right) ^3$	Emphasizes highly connected nodes
Fuzzy Sombor Index	$\sum _{(p_\alpha ,q_\alpha ) \in \beta _f} \sqrt{ \left( \mu _{\alpha _f}(p_\alpha ) \cdot \deg (p_\alpha )\right) ^2 + \left( \mu _{\alpha _f}(q_\alpha ) \cdot \deg (q_\alpha )\right) ^2 }$	Measures connectivity considering magnitude of degrees
Fuzzy Nirmala Index	$\sum _{(p_\alpha ,q_\alpha ) \in \beta _f} \sqrt{\mu _{\alpha _f}(p_\alpha )\cdot \deg (p_\alpha )+\mu _{\alpha _f}(q_\alpha )\cdot \deg (q_\alpha )}$	Measures connectivity considering magnitude of degrees

## Methodology

The methodology has been systematically developed for calculation and analysis of fuzzy The topological indices of fuzzy Helm graphs, and are shown to have applications in biological purposes. interaction networks. A classical Helm graph $HT_{\alpha }$ was constructed by appending a pendant vertex to each vertex of the outer cycle of the wheel graph. The classical structure was subsequently converted into a fuzzy Helm graph by assigning membership values in [0, 1] to both vertices and edges of the graph. Vertex membership values were assigned based on the importance of the vertices in the network, whereas edge membership values were assigned based on the strength of the corresponding interactions. To calculate the fuzzy graph descriptors, the vertices of the fuzzy Helm graph were partitioned into three groups: the central hub vertex, the cycle vertices, and the pendant vertices. The fuzzy degree of each vertex was then computed by summing the membership values of all edges incident to the vertex. Using these fuzzy degrees together with the vertex membership values, closed-form analytical expressions were derived for the following fuzzy topological indices: the fuzzy first Zagreb index, the fuzzy second Zagreb index, the fuzzy Randić index, the fuzzy Harmonic index, the fuzzy *F*-index, the fuzzy Misbalance Prodeg index, fuzzy Sombor index, and fuzzy Nirmala index. The derived formulas were checked symbolically and numerically. Substituting the general expression for the vertex and edge membership symbols into the definition of the fuzzy topological indices and algebraically simplifying the summations of these expressions was used for the symbolic verification. To check the numerical values, a concrete example of a fuzzy Helm graph, called $HT_3$, was built, and all indices were explicitly calculated. The results were then computed manually and compared with the generalized closed-form expressions to check for consistency and accuracy. The model of a biological protein–protein interaction network was constructed using a fuzzy Helm graph structure to show the applicability of the proposed framework for a biological protein–protein interaction network model. The sources of protein–protein interaction data were public databases. biological databases: the STRING database https://string-db.org/ that offers experimentally illustrated and computationally predicted protein–protein interaction confidence scores, complemented by additional functional and regulatory information from UniProtKB. The BioGRID https://thebiogrid.org/ and https://www.uniprot.org/ databases. In the biological model, P53 was identified as the central hub vertex, as it is an important molecule in the regulation of the cell. Part of its function is to regulate DNA repair, apoptosis, and cell cycle control. The strongly interacting proteins MDM2, ATM, Rb, and P21 were represented by the cycle vertices, while the secondary or weaker regulatory proteins SUMO, USP7, NF-*κ*B, and CHK2 were, represented by the pendant vertices. Fuzzy edge weights were assigned according to the interaction confidence scores obtained from STRING, with higher-confidence interactions receiving larger membership values. Vertex membership values were assigned on the basis of the biological significance and regulatory role of each protein within the P53 signaling pathway.

**Fig. 2:**
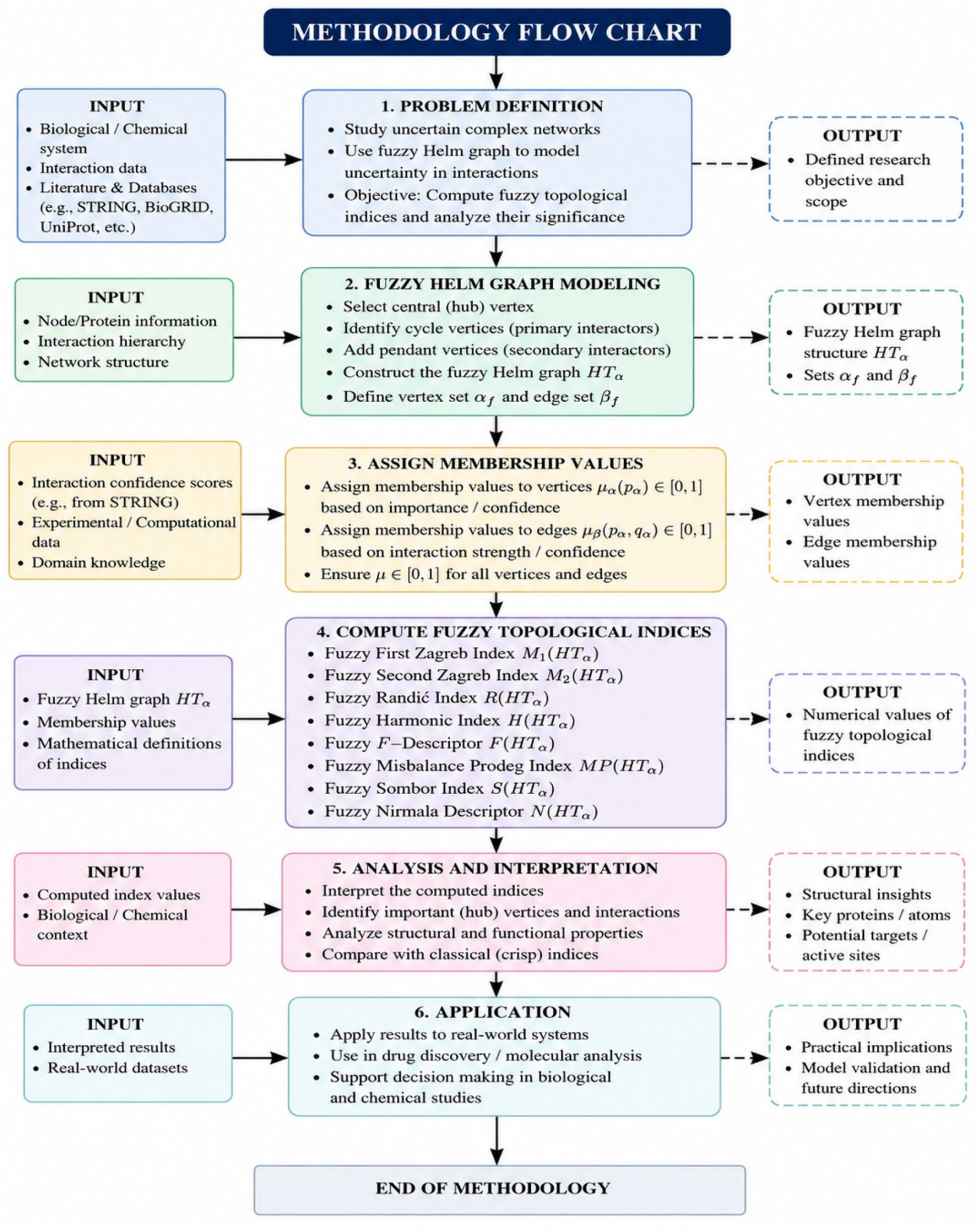
Methodology.

After constructing the fuzzy protein–protein interaction network, all proposed fuzzy Topological indices were computed numerically. The resulting values were analyzed to identify highly influential proteins, irregularities in the interaction patterns, and network behavior Under interaction uncertainty. The analysis demonstrated that fuzzy topological indices provide a richer structural interpretation than classical crisp graph descriptors, particularly for uncertain biological systems in which interactions are not strictly binary in nature. All graphical structures presented in this study were generated computationally using graph visualization tools and LaTeX-based graph drawing techniques. The methodology is illustrated in Fig. 2.

## Main results and discussion

The fuzzy Helm graph $\mathbb {H}_{T_\alpha }$ for $T_\alpha \ge 3$ is an extension of the Helm graph. In the fuzzy Helm graph $\mathbb {H}_{T_\alpha }$, the total number of vertices is $2T_\alpha +1$. $2T_\alpha$ vertices contains membership value $\frac{1}{T_\alpha }$, where remaining *1* vertex contains $\frac{1}{T^2_\alpha }$ membership value. In $\mathcal {H}_{T_\alpha }$, the total number of edges is $3T_\alpha$, in which $T_\alpha$ edges with weight $\frac{1}{T^2_\alpha }$, and $2T_\alpha$ edges contain $\frac{1}{T_\alpha }$ weight. For the degree type of the vertices, we have $T_\alpha$ pendant vertices with degree $\frac{1}{T_\alpha }$,*1* central vertex with degree $\frac{1}{T_\alpha }$, and the remaining $T_\alpha$ vertices have degree $\frac{3T_\alpha +1}{T^2_\alpha }$. For edge type with respect to end vertex degrees we have,

- $T_\alpha$ edges with end vertex weight $\frac{1}{T^2_\alpha },\frac{1}{T_\alpha }$ and end vertex degrees $\frac{1}{T_\alpha },\frac{3T_\alpha +1}{T^2_\alpha }$.
- $T_\alpha$ edges with end vertex weight $\frac{1}{T_\alpha },\frac{1}{T_\alpha }$ and end vertex degrees $\frac{3T_\alpha +1}{T^2_\alpha },\frac{3T_\alpha +1}{T^2_\alpha }$.
- $T_\alpha$ edges with end vertex weight $\frac{1}{T_\alpha },\frac{1}{T_\alpha }$ and end vertex degrees $\frac{3T_\alpha +1}{T^2_\alpha },\frac{1}{T_\alpha }$.

**Fig. 3:**
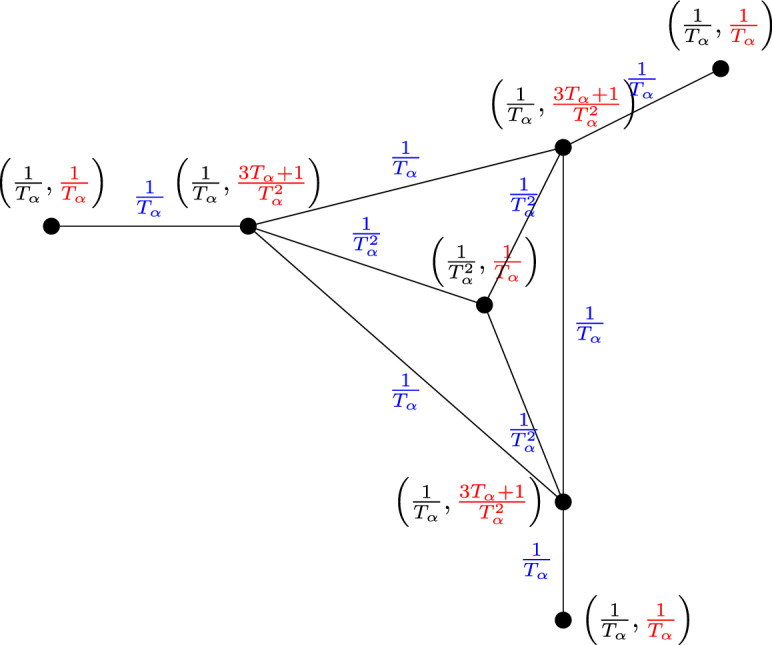
Fuzzy Helm Graph $\mathbb {H}_{T_\alpha }$.

Here, an example of $\mathbb {H}_{T_\alpha }$ for $T_\alpha =3$ is given, in which the membership values of vertices, edges, and degrees are marked with black, blue, and red colors, respectively See Fig. 3.

**Fig. 4:**
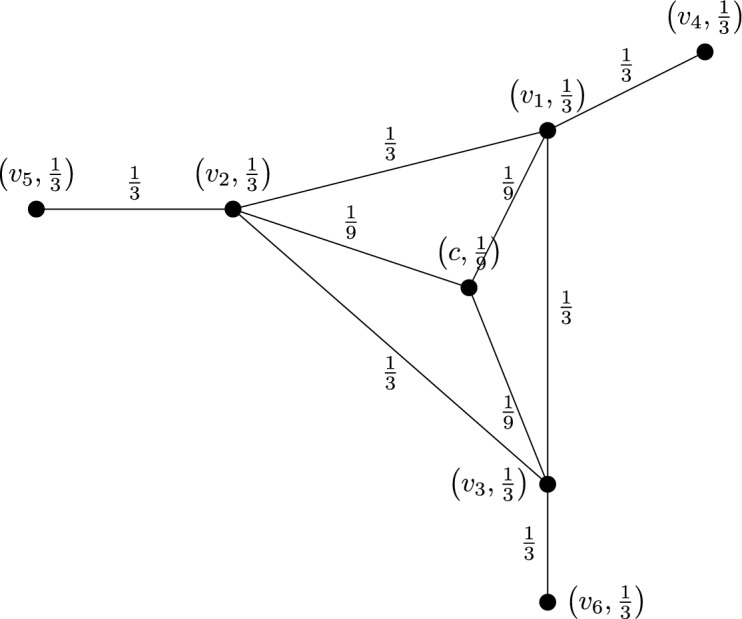
Fuzzy Helm Graph $\mathbb {H}_{T_3}$.

For $\mathbb {H}_{T_3}$, we reconsider the above-mentioned example with $T_\alpha =3$ and evaluate all the values for vertices, edges, and degrees. In Fig. 4, we have vertex set $\alpha _f=\{c,v_1,v_2,v_3,v_4,v_5,v_6\}$ with the following membership values: $\mu _{\alpha _f}(v_1)=\mu _{\alpha _f}(v_2)=\mu _{\alpha _f}(v_3)=\mu _{\alpha _f}(v_4)=\mu _{\alpha _f}(v_5)=\mu _{\alpha _f}(v_6)=\frac{1}{3},\quad and \quad \mu _{\alpha _f}(c)=\frac{1}{9}.$ For the fuzzy edge set $\beta _f=\{cv_1,cv_2,cv_3,v_1v_2,v_2v_3,v_1v_3,v_1v_4,v_2v_5,v_3v_6\}$, with membership values: $\mu _{\beta _f}(cv_1)= \mu _{\beta _f}(cv_2)=\mu _{\beta _f}(cv_2)=\frac{1}{9}$, and $\mu _{\beta _f}(v_1v_2)=\mu _{\beta _f}(v_2v_3)=\mu _{\beta _f}(v_1v_3)=\mu _{\beta _f}(v_1v_4)=\mu _{\beta _f}(v_2v_5)=\mu _{\beta _f}(v_3v_6)=\frac{1}{3}$

Fuzzy degrees are defined as the aggregate of the membership values of all edges incident to a node. The degree of each node is evaluated as follows:

$$\begin{aligned} deg(c)= & \frac{1}{9}+\frac{1}{9}+\frac{1}{9}=\frac{1}{3}, \\ deg(v_1)= & deg(v_2)=deg(v_3)=\frac{1}{3}+\frac{1}{3}+\frac{1}{3}+\frac{1}{9}=\frac{10}{9}, \\ deg(v_4)= & deg(v_5)=deg(v_6)=\frac{1}{3}. \end{aligned}$$

### Theorem 1

Let $\mathcal {H}_{T_\alpha }$ be the fuzzy Helm graph where $T_\alpha \ge 3$. The first fuzzy Zagreb index is expressed as follows:

$$\begin{aligned} \mathbb {M}_1(\mathcal {H}_{T_\alpha })= & \frac{10T^2_\alpha +6T_\alpha +2}{T^4_\alpha }, \quad \text {where }, \end{aligned}$$

$$T_\alpha \ge 3$$.

### Proof

Let the fuzzy Helm graph $\mathcal {H}_{T_\alpha }$ here $T_\alpha \ge 3$.The structure consists of $2T_\alpha +1$ nodes, $T_\alpha$ vertices along with a fuzzy node weight of $\frac{1}{T_\alpha }$ along with a degree of $\frac{1}{T_\alpha }$, also with one hub node which contains a weight of $\frac{1}{T^2_\alpha }$ and a degree of $\frac{1}{T_\alpha }$, and the remaining $T_\alpha$ vertices have a vertex weight of $\frac{1}{T_\alpha }$ with end degrees $\frac{3T_\alpha +1}{T^2_\alpha }$. Considering the fuzzy characterization of nodes, $\mathbb {M}_1(\mathcal {H}_{T_\alpha })$ can be expressed as the sum of the squares of vertex degrees weighted by their membership values:

$$\begin{aligned} \mathbb {M}_1(\mathcal {H}_{T_\alpha })= & \sum _{p_\alpha \in \alpha _f} \mu _{\alpha _f}(p_\alpha ) \cdot \left( \deg (p_\alpha ) \right) ^2 \end{aligned}$$

where $\deg (p_\alpha )$ corresponds to the degree of vertex $p_\alpha$, and $\mu _{\alpha _f}(p_\alpha )$ indicates its associated membership value. For the given fuzzy helm structure $\mathcal {H}_{T_\alpha }$, the $\mathbb {M}_1(\mathcal {H}_{T_\alpha })$ can be evaluated as

$$\begin{aligned} \mathbb {M}_1(\mathcal {H}_{T_\alpha })= & T_\alpha [\frac{1}{T_\alpha }\cdot (\frac{1}{T_\alpha })^2]+1\cdot [\frac{1}{T^2_\alpha }\cdot (\frac{1}{T_\alpha })^2]+T_\alpha [\frac{1}{T_\alpha }\cdot (\frac{3T_\alpha +1}{T^2_\alpha })^2], \end{aligned}$$

Simplifying the above expression, we get

$$\begin{aligned} \mathbb {M}_1(\mathcal {H}_{T_\alpha })= & \frac{1}{T^2_\alpha }+\frac{1}{T^4_\alpha }+\frac{(3T_\alpha +1)^2}{T^4_\alpha }, \\ \mathbb {M}_1(\mathcal {H}_{T_\alpha })= & \frac{T^2_\alpha +1+9T^2_\alpha +6T_\alpha +1}{T^4_\alpha }, \end{aligned}$$

finally we get the desired result, that is

$$\begin{aligned} \mathbb {M}_1(\mathcal {H}_{T_\alpha })= & \frac{10T^2_\alpha +6T_\alpha +2}{T^4_\alpha }. \quad \end{aligned}$$

This finalizes the proof of the theorem. *□*

### Theorem 2

For fuzzy helm graph $\mathcal {H}_{T_\alpha }$ along with $T_\alpha \ge 3$, the second fuzzy Zagreb index $\mathbb {M}_2(\mathcal {H}_{T_\alpha })$ can be formulated as

$$\begin{aligned} \mathbb {M}_2(\mathcal {H}_{T_\alpha }) = \frac{(3T_\alpha +1)(2T_\alpha +1)}{T^5_\alpha }, \end{aligned}$$

where, $$T_\alpha \ge 3$$.

### Proof

For a fuzzy helm structure $\mathcal {H}_{T_\alpha }$ with $T_\alpha \ge 3$, we consider $2T_\alpha +1$ nodes and $3T_\alpha$ edges. For this structure, $T_\alpha$ edges with end vertex weight $\frac{1}{T_\alpha },\frac{1}{T_\alpha }$ and end vertex degrees $\frac{1}{T_\alpha },\frac{3T_\alpha +1}{T^2_\alpha }$. $T_\alpha$ edges with end vertex weight $\frac{1}{T_\alpha },\frac{1}{T_\alpha }$ and end vertex degrees $\frac{3T_\alpha +1}{T^2_\alpha },\frac{3T_\alpha +1}{T^2_\alpha }$, and remaining $T_\alpha$ edges with end vertex weight $\frac{1}{T^2_\alpha },\frac{1}{T_\alpha }$ and end vertex degrees $\frac{1}{T_\alpha },\frac{3T_\alpha +1}{T^2_\alpha }$. The index $\mathbb {M}_2(\mathcal {H}_{T_\alpha })$ quantifies the fuzzy helm graph structure as half the sum of the products of the degrees and membership values of the linked nodes, accounting for node and edge fuzziness:

$$\begin{aligned} \mathbb {M}_2(\mathcal {H}_{T_\alpha })= & \frac{1}{2} \sum _{(p_\alpha ,q_\alpha ) \in \beta _f} \left( \mu _{\alpha _f}(p_\alpha ) \cdot \deg (p_\alpha ) \right) \left( \mu _{\alpha _f}(q_\alpha ) \cdot \deg (q_\alpha ) \right) , \end{aligned}$$

Here, $\deg (p_\alpha )$ and $\deg (q_\alpha )$ refer to the degrees of nodes $p_\alpha$ and $q_\alpha$, respectively. $\mu _{\alpha _f}(p_\alpha )$ and $\mu _{\alpha _f}(q_\alpha )$ denote their membership values. now, by replacing the respective values for $\mathbb {M}_2(\mathcal {H}_{T_\alpha })$, we get

$$\begin{aligned} \mathbb {M}_2(\mathcal {H}_{T_\alpha })= & T_\alpha \left[ \frac{1}{2}\left( \frac{1}{T_\alpha }\cdot \frac{1}{T_\alpha }\cdot \frac{1}{T_\alpha }\cdot \frac{3T_\alpha +1}{T^2_\alpha }\right) \right] +T_\alpha \left[ \frac{1}{2}\left( \frac{1}{T_\alpha } \cdot \frac{3T_\alpha +1}{T^2_\alpha }\cdot \frac{1}{T_\alpha }\cdot \frac{3T_\alpha +1}{T^2_\alpha }\right) \right] \\ & +T_\alpha \left[ \frac{1}{2}\left( \frac{1}{T^2_\alpha }\cdot \frac{1}{T_\alpha }\cdot \frac{1}{T_\alpha }\cdot \frac{3T_\alpha +1}{T^2_\alpha }\right) \right] , \end{aligned}$$

Simplifying, we get

$$\begin{aligned} \mathbb {M}_2(\mathcal {H}_{T_\alpha })= & \frac{T_\alpha }{2} \left( \frac{3T_\alpha +1}{T^5_\alpha } \right) +\frac{T_\alpha }{2} \left( \frac{(3T_\alpha +1)^2}{T^6_\alpha }\right) +\frac{T_\alpha }{2} \left( \frac{3T_\alpha +1}{T^6_\alpha } \right) , \\ \mathbb {M}_2(\mathcal {H}_{T_\alpha })= & \frac{T_\alpha (3T_\alpha +1)}{2T^5_\alpha }\cdot \left( 1+\frac{3T_\alpha +1}{T_\alpha }+\frac{1}{T_\alpha } \right) , \\ \mathbb {M}_2(\mathcal {H}_{T_\alpha })= & \frac{(3T_\alpha +1)}{2T^4_\alpha }\cdot \left( \frac{T_\alpha +3T_\alpha +1+1}{T_\alpha } \right) , \\ \mathbb {M}_2(\mathcal {H}_{T_\alpha })= & \left( \frac{2(3T_\alpha +1)(2T_\alpha +1)}{2T^5_\alpha } \right) , \end{aligned}$$

finally we get,

$$\begin{aligned} \mathbb {M}_2(\mathcal {H}_{T_\alpha })= & \left( \frac{(3T_\alpha +1)(2T_\alpha +1)}{T^5_\alpha } \right) . \end{aligned}$$

Hence, the theorem has been proven. *□*

### Theorem 3

For a fuzzy helm structure $\mathcal {H}_{T_\alpha }$ with $T_\alpha \ge 3$. Then, the fuzzy Randić index $\mathbb {R}(\mathcal {H}_{T_\alpha })$ is formulated as

$$\begin{aligned} \mathbb {R}(\mathcal {H}_{T_\alpha })= & \frac{T^3_\alpha }{2(3T_\alpha +1)}\left[ T_\alpha (1+\sqrt{3T_\alpha +1})+\sqrt{3T^2_\alpha +T_\alpha }\right] , \end{aligned}$$

where, $$T_\alpha \ge 3$$.

### Proof

Suppose a fuzzy Helm graph $\mathcal {H}_{T_\alpha }$ with $T_\alpha \ge 3$, which consists of $2T_\alpha + 1$ vertices and $3T_\alpha$ edges. The graph can be analyzed based on the types of edges, corresponding degrees, and membership values of the vertices. $T_\alpha$ edges with end vertex weight $\frac{1}{T_\alpha },\frac{1}{T_\alpha }$ and end vertex degrees $\frac{1}{T_\alpha },\frac{3T_\alpha +1}{T^2_\alpha }$. $T_\alpha$ edges with end vertex weight $\frac{1}{T_\alpha },\frac{1}{T_\alpha }$ and end vertex degrees $\frac{3T_\alpha +1}{T^2_\alpha },\frac{3T_\alpha +1}{T^2_\alpha }$, and the remaining $T_\alpha$ edges with end vertex weight $\frac{1}{T^2_\alpha },\frac{1}{T_\alpha }$ and end vertex degrees $\frac{1}{T_\alpha },\frac{3T_\alpha +1}{T^2_\alpha }$. The descriptor $\mathbb {R}(\mathcal {H}_{T_\alpha })$ is elaborated as

$$\begin{aligned} \mathbb {R}(\mathcal {H}_{T_\alpha })= & \frac{1}{2} \sum _{(p_\alpha ,q_\alpha ) \in \beta _f} \frac{1}{\sqrt{\mu _{\alpha _f}(p_\alpha ) \cdot \deg (p_\alpha ) \cdot \mu _{\alpha _f}(q_\alpha ) \cdot \deg (q_\alpha ) }}. \end{aligned}$$

With respect to the first type of edges, the $T_\alpha$ edges of this type have end nodes with membership values $\frac{1}{T_\alpha },\frac{1}{T_\alpha }$ and end degree $\frac{1}{T_\alpha } ,\frac{3T_\alpha +1}{T^2_\alpha }$. For the next class, $T_\alpha$ edges have endpoints with membership values$\frac{1}{T_\alpha },\frac{1}{T_\alpha }$, and the degrees of the end nodes are $\frac{3T_\alpha +1}{T^2_\alpha },\frac{3T_\alpha +1}{T^2_\alpha }$. The remaining edges $T_\alpha$ have membership values $\frac{1}{T_\alpha },\frac{1}{T_\alpha }$, along with end degrees $\frac{1}{T_\alpha },\frac{3T_\alpha +1}{T^2_\alpha }$. The portion of the index corresponding to these edges is

$$\begin{aligned} \mathbb {R}(\mathcal {H}_{T_\alpha })= & \frac{T_\alpha }{2}\left[ \frac{1}{\sqrt{\frac{1}{T_\alpha }\frac{1}{T_\alpha }\cdot \frac{1}{T_\alpha }\frac{3T_\alpha +1}{T^2_\alpha }}}\right] +\frac{T_\alpha }{2}\left[ \frac{1}{\sqrt{\frac{1}{T^2_\alpha }\frac{1}{T_\alpha }\cdot \frac{1}{T_\alpha }\frac{3T_\alpha +1}{T^2_\alpha }}}\right] \\ & +\frac{T_\alpha }{2}\left[ \frac{1}{\sqrt{\frac{1}{T_\alpha }\frac{3T_\alpha +1}{T^2_\alpha }\cdot \frac{1}{T_\alpha }\frac{3T_\alpha +1}{T^2_\alpha }}}\right] , \end{aligned}$$

The expression is simplified as follows:

$$\begin{aligned} \mathbb {R}(\mathcal {H}_{T_\alpha })= & \frac{T_\alpha }{2}\left[ \frac{1}{\sqrt{\frac{3T_\alpha +1}{T^5_\alpha }}}\right] +\frac{T_\alpha }{2}\left[ \frac{1}{\sqrt{\frac{3T_\alpha +1}{T^6_\alpha }}}\right] +\frac{T_\alpha }{2}\left[ \frac{1}{\sqrt{\frac{(3T_\alpha +1)^2}{T^6_\alpha }}}\right] ,\\ \mathbb {R}(\mathcal {H}_{T_\alpha })= & \frac{T_\alpha }{2}\left[ \frac{T^2_\alpha \sqrt{T_\alpha }}{\sqrt{3T_\alpha +1}}\right] +\frac{T_\alpha }{2}\left[ \frac{T^3_\alpha }{\sqrt{3T_\alpha +1}}\right] +\frac{T_\alpha }{2}\left[ \frac{T^3_\alpha }{3T_\alpha +1}\right] , \\ \mathbb {R}(\mathcal {H}_{T_\alpha })= & \frac{T^3_\alpha }{2\sqrt{3T_\alpha +1}}\left[ \frac{\sqrt{T_\alpha }\sqrt{3T_\alpha +1}+T_\alpha +T_\alpha \sqrt{3T_\alpha +1}}{\sqrt{3T_\alpha +1}} \right] , \end{aligned}$$

so, the final expression we get is

$$\begin{aligned} \mathbb {R}(\mathcal {H}_{T_\alpha })= & \frac{T^3_\alpha }{2(3T_\alpha +1)}\left[ T_\alpha (1+\sqrt{3T_\alpha +1})+\sqrt{3T^2_\alpha +T_\alpha }\right] . \end{aligned}$$

This concludes the proof. *□*

### Theorem 4

Let us consider a fuzzy Helm graph $\mathcal {H}_{T_\alpha }$ with $T_\alpha \ge 3$. Then the fuzzy Harmonic index $\mathbb {H}(\mathcal {H}_{T_\alpha })$ can be written as

$$\begin{aligned} \mathbb {H}(\mathcal {H}_{T_\alpha })= & \frac{T^4_\alpha }{2}\left[ \frac{1}{4T_\alpha +1}+\frac{1}{3T_\alpha +2}+\frac{1}{2(3T_\alpha +1)}\right] , \end{aligned}$$

where, $$T_\alpha \ge 3$$.

### Proof

We examine the fuzzy helm graph $\mathcal {H}_{T_\alpha }$ with $T_\alpha \ge 3$, which contains $2T_\alpha +1$ nodes and $3T_\alpha$ edges. We describe the structural features of this graph as follows: $T_\alpha$ edges with end vertex weight $\frac{1}{T_\alpha },\frac{1}{T_\alpha }$ and end vertex degrees $\frac{1}{T_\alpha },\frac{3T_\alpha +1}{T^2_\alpha }$. $T_\alpha$ edges with end vertex weight $\frac{1}{T_\alpha },\frac{1}{T_\alpha }$ and end vertex degrees $\frac{3T_\alpha +1}{T^2_\alpha },\frac{3T_\alpha +1}{T^2_\alpha }$, and remaining $T_\alpha$ edges with end vertex weight $\frac{1}{T^2_\alpha },\frac{1}{T_\alpha }$ and end vertex degrees $\frac{1}{T_\alpha },\frac{3T_\alpha +1}{T^2_\alpha }$. The $\mathbb {H}(\mathcal {H}_{T_\alpha })$ is given as

$$\begin{aligned} \mathbb {H}(\mathcal {H}_{T_\alpha })= & \frac{1}{2} \sum _{(p_\alpha ,q_\alpha ) \in \beta _f} \frac{1}{\mu _{\alpha _f}(p_\alpha ) \cdot \deg (p_\alpha ) + \mu _{\alpha _f}(q_\alpha ) \cdot \deg (q_\alpha )}, \end{aligned}$$

Using the membership values and degrees for three distinct types of edges, we have

$$\begin{aligned} \mathbb {H}(\mathcal {H}_{T_\alpha })= & \frac{T_\alpha }{2}\left[ \frac{1}{\frac{1}{T_\alpha }\frac{1}{T_\alpha }+\frac{1}{T_\alpha }\frac{3T_\alpha +1}{T^2_\alpha }}\right] +\frac{T_\alpha }{2}\left[ \frac{1}{\frac{1}{T^2_\alpha }\frac{1}{T_\alpha }+\frac{1}{T_\alpha }\frac{3T_\alpha +1}{T^2_\alpha }}\right] \\ & +\frac{T_\alpha }{2}\left[ \frac{1}{\frac{1}{T_\alpha }\frac{3T_\alpha +1}{T^2_\alpha }+\frac{1}{T_\alpha }\frac{3T_\alpha +1}{T^2_\alpha }}\right] , \end{aligned}$$

simplify the given expression

$$\begin{aligned} \mathbb {H}(\mathcal {H}_{T_\alpha })= & \frac{T_\alpha }{2}\left[ \frac{1}{\frac{1}{T^2_\alpha }+\frac{3T_\alpha +1}{T^3_\alpha }}\right] +\frac{T_\alpha }{2}\left[ \frac{1}{\frac{1}{T^3_\alpha }+\frac{3T_\alpha +1}{T^3_\alpha }}\right] +\frac{T_\alpha }{2}\left[ \frac{1}{\frac{3T_\alpha +1}{T^3_\alpha }+\frac{3T_\alpha +1}{T^3_\alpha }}\right] , \end{aligned}$$

after further simplification,

$$\begin{aligned} \mathbb {H}(\mathcal {H}_{T_\alpha })= & \frac{T_\alpha }{2}\left[ \frac{T^3_\alpha }{T_\alpha +3T_\alpha +1}\right] +\frac{T_\alpha }{2}\left[ \frac{T^3_\alpha }{1+3T_\alpha +1}\right] +\frac{T_\alpha }{2}\left[ \frac{T^3_\alpha }{3T_\alpha +1+3T_\alpha +1}\right] , \end{aligned}$$

so, finally we get

$$\begin{aligned} \mathbb {H}(\mathcal {H}_{T_\alpha })= & \frac{T^4_\alpha }{2}\left[ \frac{1}{4T_\alpha +1}+\frac{1}{3T_\alpha +2}+\frac{1}{2(3T_\alpha +1)}\right] . \end{aligned}$$

This completes the theorem’proof. *□*

### Theorem 5

For a fuzzy Helm graph $\mathcal {H}_{T_\alpha }$ with $T_\alpha \ge 3$. The fuzzy F descriptor for fuzzy Helm graph $\mathbb {F}(\mathcal {H}_{T_\alpha })$ is formulated as

$$\begin{aligned} \mathbb {F}(\mathcal {H}_{T_\alpha })= & \frac{T^4_\alpha +1+T_\alpha (3T_\alpha +1)^3}{T^9_\alpha } \end{aligned}$$

where $$T_\alpha \ge 3$$.

### Proof

Given $\mathcal {H}_{T_\alpha }$ is a fuzzy Helm graph with $2T_\alpha +1$ vertices. The graph consists of $2T_\alpha +1$ vertices, $T_\alpha$ vertices with a fuzzy vertex weight of $\frac{1}{T_\alpha }$ and a degree of $\frac{1}{T_\alpha }$, along with one hub node which contains value of $\frac{1}{T^2_\alpha }$ and a degree of $\frac{1}{T_\alpha }$, and the remaining $T_\alpha$ vertices have a vertex weight of $\frac{1}{T_\alpha }$ with end degrees $\frac{3T_\alpha +1}{T^2_\alpha }$.

$$\begin{aligned} \mathbb {F}(\mathcal {H}_{T_\alpha })= & \sum _{p_\alpha \in \alpha _f} \left( \mu _{\alpha _f}(p_\alpha ) \cdot \deg (p_\alpha )\right) ^3 \end{aligned}$$

The $2T_\alpha +1$ nodes contribute to the fuzzy F descriptor as

$$\begin{aligned} \mathbb {F}(\mathcal {H}_{T_\alpha })= & T_\alpha \left[ \frac{1}{T_\alpha } \cdot \frac{1}{T_\alpha }\right] ^3+\left[ \frac{1}{T^2_\alpha } \cdot \frac{1}{T_\alpha }\right] ^3+T_\alpha \left[ \frac{1}{T_\alpha } \cdot \frac{3T_\alpha +1}{T^2_\alpha }\right] ^3, \end{aligned}$$

simplify the terms:

$$\begin{aligned} \mathbb {F}(\mathcal {H}_{T_\alpha })= & T_\alpha \left[ \frac{1}{T^6_\alpha }\right] +\left[ \frac{1}{T^9_\alpha }\right] +T_\alpha \left[ \frac{(3T_\alpha +1)^3}{T^9_\alpha }\right] , \\= & \frac{1}{T^5_\alpha }+\frac{1}{T^9_\alpha }+\frac{(3T_\alpha +1)^3}{T^8_\alpha }, \end{aligned}$$

after further simplification

$$\begin{aligned} \mathbb {F}(\mathcal {H}_{T_\alpha })= & \frac{T^4_\alpha +1+T_\alpha (3T_\alpha +1)^3}{T^9_\alpha }. \end{aligned}$$

This completes the proof. *□*

### Theorem 6

Let us consider a fuzzy Helm graph $\mathcal {H}_{T_\alpha }$ with $T_\alpha \ge 3$. The fuzzy Misbalance Prodeg index $\mathbb{M}\mathbb{P}(\mathcal {H}_{T_\alpha })$ for this structure is formulated as

$$\begin{aligned} \mathbb{M}\mathbb{P}(\mathcal {H}_{T_\alpha })= & 1+\frac{1}{\sqrt{T_\alpha }}+4\sqrt{\frac{3T_\alpha +1}{T_\alpha }} \end{aligned}$$

where $$T_\alpha \ge 3$$.

### Proof

The fuzzy Misbalance Prodeg index $\mathbb{M}\mathbb{P}(H_{T_\alpha )}$ for a fuzzy Helm graph $\mathcal {H}_{T_\alpha }$ is defined as

$$\begin{aligned} \mathbb{M}\mathbb{P}(\mathcal {H}_{T_\alpha })= & \sum _{(p_\alpha ,q_\alpha ) \in \beta _f} \left( \sqrt{\mu _{\alpha _f}(p_\alpha )\cdot \deg (p_\alpha )} + \sqrt{\mu _{\alpha _f}(q_\alpha )\cdot \deg (q_\alpha )}\right) \end{aligned}$$

Given a fuzzy Helm graph $\mathcal {H}_{T_\alpha }$ with the following properties: $T_\alpha$ edges with end degree type $\left( \frac{1}{T_\alpha }, \frac{3T_\alpha +1}{T^2_\alpha }\right)$ and end vertices have membership values $\left( \frac{1}{T_\alpha }, \frac{1}{T_\alpha }\right)$, $T_\alpha$ edges with end degrees $\left( \frac{1}{T_\alpha }\right)$ and corresponding end vertex membership values $\frac{3T_\alpha +1}{T^2_\alpha }$. remaining $T_\alpha$ edges have end degree type $\frac{1}{T_\alpha },\frac{3T_\alpha +1}{T_\alpha }$ with vertex membership values $\frac{1}{T^2_\alpha },\frac{1}{T_\alpha }$. We evaluate the contribution of each category of edges to the fuzzy Misbalance prodeg index as follow

$$\begin{aligned} \mathbb{M}\mathbb{P}(\mathcal {H}_{T_\alpha })= & T_\alpha \left[ \sqrt{\frac{1}{T_\alpha }\cdot \frac{1}{T_\alpha }}+\sqrt{\frac{1}{T_\alpha }\cdot \frac{3T_\alpha +1}{T^2_\alpha }}\right] +T_\alpha \left[ \sqrt{\frac{1}{T_\alpha }\cdot \frac{3T_\alpha +1}{T^2_\alpha }}+\sqrt{\frac{1}{T_\alpha }\cdot \frac{3T_\alpha +1}{T^2_\alpha }} \right] \\ & T_\alpha \left[ \sqrt{\frac{1}{T^2_\alpha }\cdot \frac{1}{T_\alpha }}+\sqrt{\frac{1}{T_\alpha }\cdot \frac{3T_\alpha +1}{T^2_\alpha }} \right] \end{aligned}$$

now, simplify the given expression:

$$\begin{aligned} \mathbb{M}\mathbb{P}(\mathcal {H}_{T_\alpha })= & T_\alpha \left[ \frac{1}{T_\alpha }+\frac{\sqrt{3T_\alpha +1}}{T_\alpha \sqrt{T_\alpha }}\right] +T_\alpha \left[ \frac{2\sqrt{3T_\alpha +1}}{T_\alpha \sqrt{T_\alpha }}\right] +T_\alpha \left[ \frac{1}{T_\alpha \sqrt{T_\alpha }}+\frac{\sqrt{3T_\alpha +1}}{T_\alpha \sqrt{T_\alpha }}\right] \end{aligned}$$

after further simplification, we obtain

$$\begin{aligned} \mathbb{M}\mathbb{P}(\mathcal {H}_{T_\alpha })= & 1+\frac{1}{\sqrt{T_\alpha }}+4\sqrt{\frac{3T_\alpha +1}{T_\alpha }} \end{aligned}$$

Accordingly, the theorem has been proven. *□*

### Theorem 7

For a fuzzy Helm graph $\mathcal {H}_{T_\alpha }$ with $T_\alpha \ge 3$. The fuzzy Sombor index $\mathbb {S}(\mathcal {H}_{T_\alpha })$ is expressed as

$$\begin{aligned} \mathbb {S}(\mathcal {H}_{T_\alpha })= & \frac{1}{T^2_\alpha }\left[ \sqrt{10T^2_\alpha +6T_\alpha +1}+\sqrt{2}(3T_\alpha +1)+\sqrt{9T^2_\alpha +6T_\alpha +2}\right] \end{aligned}$$

### Proof

We examine the fuzzy Helm graph $\mathcal {H}_{T_\alpha }$, which contains $2T_\alpha +1$ nodes and exhibits the following characteristics: $T_\alpha$ nodes, each endowed with a fuzzy vertex value of $\frac{1}{T_\alpha }$ along with end degree of $\frac{1}{T_\alpha }$. One central vertex with a fuzzy vertex weight of $\frac{1}{T_\alpha ^2}$ and a degree of $\frac{1}{T_\alpha }$, and the remaining $T_\alpha$ vertices have membership values of $\frac{1}{T_\alpha }$ with end degrees $\frac{3T_\alpha +1}{T^2_\alpha }$. $3T_\alpha$ are total edges, with $T_\alpha$ edges whose nodes possess membership values $\left( \frac{1}{T_\alpha }, \frac{1}{T_\alpha }\right)$ and end vertex degrees $\left( \frac{1}{T_\alpha }, \frac{3T_\alpha +1}{T^2_\alpha }\right)$. $T_\alpha$ edges with end nodes with degree type $\left( \frac{3T_\alpha +1}{T^2_\alpha },\frac{3T_\alpha +1}{T^2_\alpha }\right)$ and vertex membership values $\left( \frac{1}{T_\alpha }, \frac{1}{T_\alpha }\right)$. All other $T_\alpha$ edges have vertex membership values $\frac{1}{T^2_\alpha },\frac{1}{T_\alpha }$ with end degrees $\frac{1}{T_\alpha },\frac{3T_\alpha +1}{T^2_\alpha }$. The $\mathbb {S}(\mathcal {H}_{T_\alpha })$ for a fuzzy structure $\mathcal {H}_{T_\alpha } = (\alpha _f, \beta _f)$ is expressed as

$$\begin{aligned} \mathbb {S}(\mathcal {H}_{T_\alpha })= & \sum _{(p_\alpha ,q_\alpha ) \in \beta _f} \sqrt{ \left( \mu _{\alpha _f}(p_\alpha )\cdot \deg (p_\alpha )\right) ^2 + \left( \mu _{\alpha _f}(q_\alpha )\cdot \deg (q_\alpha )\right) ^2 } \end{aligned}$$

We evaluate this sum by replacing the respective values as follows:

$$\begin{aligned} \mathbb {S}(\mathcal {H}_{T_\alpha })= & T_\alpha \sqrt{ \left( \frac{1}{T_\alpha } \cdot \frac{1}{T_\alpha }\right) ^2 + \left( \frac{1}{T_\alpha } \cdot \frac{3T_\alpha +1}{T^2_\alpha }\right) ^2 }+T_\alpha \sqrt{ \left( \frac{1}{T_\alpha } \cdot \frac{3T_\alpha +1}{T^2_\alpha }\right) ^2 + \left( \frac{1}{T_\alpha } \cdot \frac{3T_\alpha +1}{T^2_\alpha }\right) ^2 } \\ & + T_\alpha \sqrt{ \left( \frac{1}{T^2_\alpha } \cdot \frac{1}{T_\alpha }\right) ^2 + \left( \frac{1}{T_\alpha } \cdot \frac{3T_\alpha +1}{T^2_\alpha }\right) ^2 } \end{aligned}$$

Simplifying,

$$\begin{aligned} \mathbb {S}(\mathcal {H}_{T_\alpha })= & T_\alpha \left[ \sqrt{\frac{1}{T^4_\alpha }+\frac{(3T_\alpha +1)^2}{T^6_\alpha }}\right] +\sqrt{2}T_\alpha \left[ \frac{3T_\alpha +1}{T^3_\alpha }\right] \\ & +T_\alpha \left[ \sqrt{\frac{1}{T^6_\alpha }+\frac{(3T_\alpha +1)^2}{T^6_\alpha }}\right] \end{aligned}$$

further Simplifying,

$$\begin{aligned} \mathbb {S}(\mathcal {H}_{T_\alpha })= & T_\alpha \left[ \sqrt{\frac{T_\alpha +(3T_\alpha +1)^2}{T^6_\alpha }}\right] +\left[ \sqrt{2}\frac{(3T_\alpha +1)}{T^2_\alpha }\right] +T_\alpha \left[ \sqrt{\frac{1+(3T_\alpha +1)^2}{T^6_\alpha }}\right] \end{aligned}$$

Finally, the total fuzzy Sombor index $\mathbb {S}(\mathcal {H}_{T_\alpha })$ is evaluated as follows:

$$\begin{aligned} \mathbb {S}(\mathcal {H}_{T_\alpha })= & \frac{1}{T^2_\alpha }\left[ \sqrt{10T^2_\alpha +6T_\alpha +1}+\sqrt{2}(3T_\alpha +1)+\sqrt{9T^2_\alpha +6T_\alpha +2}\right] \end{aligned}$$

This concludes the proof of the theorem. *□*

### Theorem 8

The fuzzy Nirmala descriptor $\mathbb {N}(\mathcal {H}_{T_\alpha })$ where $T_\alpha \ge 3$, for fuzzy Helm structure $\mathcal {H}_{T_\alpha }$is provided as

$$\begin{aligned} \mathbb {N}(\mathcal {H}_{T_\alpha })= & \sqrt{\frac{4T_\alpha +1}{T_\alpha }}+\sqrt{\frac{2(3T_\alpha +1)}{T_\alpha }}+\sqrt{\frac{3T_\alpha +2}{T_\alpha }} \end{aligned}$$

where $$T_\alpha \ge 3$$.

### Proof

Let us consider the fuzzy Helm graph $\mathcal {H}_{T_\alpha }$ with $T_\alpha \ge 3$, possessing $2T_\alpha +1$ nodes and $3T_\alpha$ edges. The structure of the graph can be detailed as follows: $T_\alpha$ edges with end vertex weight $\frac{1}{T_\alpha },\frac{1}{T_\alpha }$ and end vertex degrees $\frac{1}{T_\alpha },\frac{3T_\alpha +1}{T^2_\alpha }$. $T_\alpha$ edges with end vertex weight $\frac{1}{T_\alpha },\frac{1}{T_\alpha }$ and end vertex degrees $\frac{3T_\alpha +1}{T^2_\alpha },\frac{3T_\alpha +1}{T^2_\alpha }$, and remaining $T_\alpha$ edges with end vertex weight $\frac{1}{T^2_\alpha },\frac{1}{T_\alpha }$ and end vertex degrees $\frac{1}{T_\alpha },\frac{3T_\alpha +1}{T^2_\alpha }$. fuzzy Nirmala descriptor $\mathbb {N}(\mathcal {H}_{T_\alpha })$ can be formulated as

$$\begin{aligned} \mathbb {N}(\mathcal {H}_{T_\alpha })= & \sum _{(p_\alpha ,q_\alpha ) \in \beta _f} \sqrt{\mu _{\alpha _f}(p_\alpha ) \cdot \deg (p_\alpha ) + \mu _{\alpha _f}(q_\alpha ) \cdot \deg (q_\alpha )} \end{aligned}$$

to compute the fuzzy Nirmala descriptor $\mathbb {N}(\mathcal {H}_{T_\alpha })$. For total $3T_\alpha$ edges, we account for the contributions arising from the given classes of edges in $\mathcal {H}_{T_\alpha }$ $T_\alpha$ edges with end vertex weight $\frac{1}{T_\alpha },\frac{1}{T_\alpha }$ and end vertex degrees $\frac{1}{T_\alpha },\frac{3T_\alpha +1}{T^2_\alpha }$. $T_\alpha$ edges with end vertex weight $\frac{1}{T_\alpha },\frac{1}{T_\alpha }$ and end vertex degrees $\frac{3T_\alpha +1}{T^2_\alpha },\frac{3T_\alpha +1}{T^2_\alpha }$, and remaining $T_\alpha$ edges with end vertex weight $\frac{1}{T^2_\alpha },\frac{1}{T_\alpha }$ and end vertex degrees $\frac{1}{T_\alpha },\frac{3T_\alpha +1}{T^2_\alpha }$. Now, calculate the fuzzy Nirmala index $\mathbb {N}(\mathcal {H}_{T_\alpha })$ for each edge type:

$$\begin{aligned} \mathbb {N}(\mathcal {H}_{T_\alpha })= & T_\alpha \sqrt{\frac{1}{T_\alpha }\cdot \frac{1}{T_\alpha } + \frac{1}{T_\alpha } \cdot \frac{3T_\alpha +1}{T^2_\alpha }}+T_\alpha \sqrt{\frac{1}{T_\alpha }\cdot \frac{3T_\alpha +1}{T^2_\alpha } + \frac{1}{T_\alpha } \cdot \frac{3T_\alpha +1}{T^2_\alpha }} \\ & +T_\alpha \sqrt{\frac{1}{T^2_\alpha }\cdot \frac{1}{T_\alpha } + \frac{1}{T_\alpha } \cdot \frac{3T_\alpha +1}{T^2_\alpha }} \end{aligned}$$

after simplification:

$$\begin{aligned} \mathbb {N}(\mathcal {H}_{T_\alpha })= & T_\alpha \left[ \sqrt{\frac{1}{T^2_\alpha }+\frac{3T_\alpha +1}{T^3_\alpha }}\right] +\sqrt{2}T_\alpha \left[ \sqrt{\frac{3T_\alpha +1}{T^3_\alpha }}\right] +T_\alpha \left[ \sqrt{\frac{1}{T^3_\alpha }+\frac{3T_\alpha +1}{T^3_\alpha }}\right] \end{aligned}$$

after further simplification,

$$\begin{aligned} \mathbb {N}(\mathcal {H}_{T_\alpha })= & T_\alpha \left[ \sqrt{\frac{T_\alpha +3T_\alpha +1}{T^3_\alpha }}\right] +\sqrt{2}T_\alpha \left[ \sqrt{\frac{3T_\alpha +1}{T^3_\alpha }}\right] +T_\alpha \left[ \sqrt{\frac{1+3T_\alpha +1}{T^3_\alpha }}\right] \end{aligned}$$

finally we get:

$$\begin{aligned} \mathbb {N}(\mathcal {H}_{T_\alpha })= & \sqrt{\frac{4T_\alpha +1}{T_\alpha }}+\sqrt{\frac{2(3T_\alpha +1)}{T_\alpha }}+\sqrt{\frac{3T_\alpha +2}{T_\alpha }} \end{aligned}$$

This concludes the proof of the theorem. *□*

### Numerical verification of derived formulas

To verify the derived closed-form expressions, we compute all fuzzy topological indices for the fuzzy Helm graph $\mathcal {H}_{T_\alpha }$ for $T_\alpha = 3$ and compare them with direct computation using the membership values and degrees shown in Fig. 4.

For $T_\alpha = 3$, the closed-form expressions are

**Table 2:** Numerical verification of fuzzy topological indices for $\mathcal {H}_{T_3}$.

Index	Closed-form value at *T=3*	Direct computation from Figure 3
$\mathbb {M}_1$	$\frac{10(3)^2+6(3)+2}{3^4} =\frac{90+18+2}{81} =\frac{110}{81} \approx 1.3580$	$3\left[ \frac{1}{3}\left( \frac{1}{3}\right) ^2\right] +\frac{1}{9}\left( \frac{1}{3}\right) ^2 +3\left[ \frac{1}{3}\left( \frac{10}{9}\right) ^2\right] =\frac{110}{81}$
$\mathbb {M}_2$	$\frac{(10)(7)}{3^5} =\frac{70}{243} \approx 0.2881$	$$\begin{aligned} & \frac{3}{2}\left( \frac{1}{3}\cdot \frac{1}{3}\cdot \frac{1}{3}\cdot \frac{10}{9}\right) +\frac{3}{2}\left( \frac{1}{3}\cdot \frac{10}{9}\cdot \frac{1}{3}\cdot \frac{10}{9}\right)\\&\quad +\frac{3}{2}\left( \frac{1}{9}\cdot \frac{1}{3}\cdot \frac{1}{3}\cdot \frac{10}{9}\right) =\frac{70}{243}\end{aligned}$$
$\mathbb {R}$	$\frac{27}{2(10)}[3(1+\sqrt{10})+\sqrt{27+3}]=\frac{27}{20}(3+3\sqrt{10}+\sqrt{30}) \approx 24.2515$	Direct computation from edge partition yields same value
$\mathbb {H}$	$\frac{81}{2}\left[ \frac{1}{13}+\frac{1}{11}+\frac{1}{20}\right] =40.5(0.07692+0.09091+0.05000) \approx 8.8222$	Direct computation from edge partition yields same value
$\mathbb {F}$	$\frac{81+1+3(10)^3}{3^9} =\frac{81+1+3000}{19683} =\frac{3082}{19683} \approx 0.1566$	Vertex-based sum yields same value
$\mathbb{M}\mathbb{P}$	$1+\frac{1}{\sqrt{3}}+4\sqrt{\frac{10}{3}} \approx 1+0.5774+7.303=8.8803$	Edge-based sum yields same value
$\mathbb {S}$	$\frac{1}{9}[\sqrt{90+18+1}+\sqrt{2}(10)+\sqrt{81+18+2}]=\frac{1}{9}[\sqrt{109}+10\sqrt{2}+\sqrt{101}] \approx 3.8480$	Edge-based sum yields same value
$\mathbb {N}$	$\sqrt{\frac{13}{3}}+\sqrt{\frac{20}{3}}+\sqrt{\frac{11}{3}} =\frac{1}{\sqrt{3}}(\sqrt{13}+\sqrt{20}+\sqrt{11}) \approx 6.5785$	Edge-based sum yields same value

The table confirms that the closed-form formulas produce values identical to the direct edge-by-edge computation using the fuzzy membership values and degrees shown in Fig. 4.

These findings show that fuzzy topological descriptors successfully reflect the structural properties of the fuzzy Helm graph by incorporating uncertainty in vertex and edge connections. Compared to crisp indices, fuzzy indices, such as fuzzy Zagreb, Randić, Harmonic, and misbalance prodeg indices, offer deeper insights, especially in networks with imprecise interactions, such as the *P*53 protein network. These indices are sensitive to changes in the edge membership values and can therefore be used to study real-world uncertain systems. This study illustrates the use of fuzzy graph-based approaches for better structural interpretation and provides new opportunities for applications in network science and biology.

## A practical application of fuzzy Helm graphs in protein networks

The presented P53 interaction network is an illustrative fuzzy graph network (model) showing the use of fuzzy Helm graph descriptors in biological network modeling. Protein interaction networks (PINs) play a vital role in understanding cellular functions, disease mechanisms, and drug-target interactions. However, there are uncertainties in protein interactions because of the experimental conditions and different binding affinities. These uncertainties can be successfully represented in a fuzzy Helm graph if protein-protein interactions are assigned fuzzy values. Moreover, topological indices, such as the fuzzy Zagreb indices, fuzzy Randić index, Fuzzy Sombor index, fuzzy Harmonic index, and others, can be used to quantify the structural characteristics and be applied in the process of drug discovery. The construction of a fuzzy Helm graph for a given protein interaction network is performed as follows:

- **Central Node:** Represents a hub protein(e.g., a regulatory protein such as ${\textbf {P}}53$ in cancer pathways)
- **Cycle nodes:**Represent proteins that directly interact with the central protein.
- **Pendant nodes:** Represent secondary (weak) interactions and/or transient protein associations.
- **Fuzzy Edge Weights:** A fuzzy weight is assigned to each interaction to represent the uncertainty of interaction.

### *P*53 cancer pathway as a fuzzy Helm graph

**Fig. 5:**
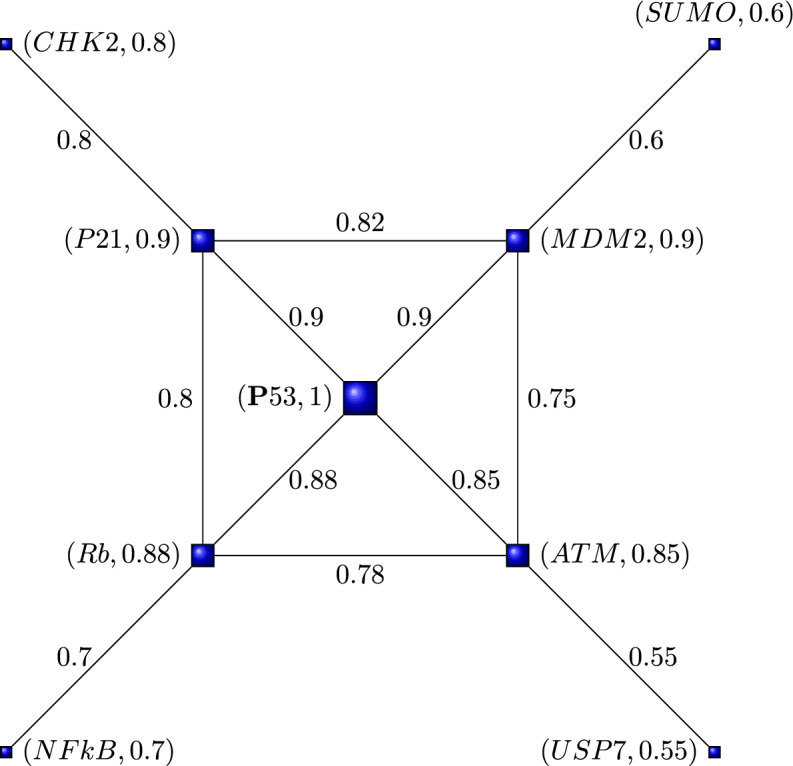
*P*53 Cancer Pathway as a Fuzzy Helm Graph.

*P*53 is a tumor suppressor protein that is a master regulator of cell arrest, DNA repair, and apoptosis. To model its interaction network under uncertainty, it is represented as a fuzzy Helm graph in Figure 5 where:

- The central vertex is *P*53 (Tumor Protein 53), which has a regulatory role and is an important protein that regulates the cell cycle, repairs DNA, and induces apoptosis. A fuzzy weight of 1 was used to indicate high confidence in the function of a central hub.
- Primary Interacting Proteins (Cycle Vertices): These proteins such as *MDM*2, *ATM*, *Rb*, and *P*21 form a cycle around *P*53 in the fuzzy Helm graph.
- *MDM* **2** (Mouse Double Minute): Regulator of degradation of *P*53. The high interaction weight is 0.9, which represents a strong negative feedback loop.
- Activation of *ATM* (Ataxia-Telangiectasia Mutated): In response to DNA damage, it activates *P*53. A moderate interaction weight 0.85 is assigned owing to activation under stress conditions.
- *Rb* (Retinoblastoma): Works alongside *P*53 in cell cycle regulation. The fuzzy weight 0.88 shows its importance in the *P*53 pathway.
- *P*21 (Cyclin-Dependent Kinase Inhibitor): One of the targets of *P*53 responsible for cell cycle arrest. The high-confidence association weight 0.9 suggests that it is directly linked to the activity of *P*53.
- Peripheral Regulatory Proteins (Pendant Vertices): The nodes *SUMO*, *USP*7, *NF*-*kB* and *CHK*2 are secondary regulatory proteins attached to the vertices of the cycle and which regulate *P*53 activity. Every interaction (edge) is assigned a fuzzy weight that reflects the level of association confidence, experimental confidence score, or level of protein expression.
- *SUMO* Small Ubiquitin-like Modifier (SUMO): Associated with *MDM*2 and modifies *MDM*2 affecting the degradation of *P*53. This is considered a secondary role; thus, a lower fuzzy weight 0.6 is assigned.
- *USP* **7** Ubiquitin-Specific Peptidase 7: Associated with *ATM* and indirectly involved in the deubiquitinating pathways of *P*53 stabilization.
- *NF***-***kB* (Nuclear Factor Kappa B): Associated with *Rb*, a transcription factor that regulates inflammation and cell survival. The fuzzy weight 0.7 is its conditional role in the stress response.
- *CHK* **2** (Checkpoint Kinase 2): Connected to *P*21 and involved in the DNA damage response. It controls the activation of gene *P*53. The fuzzy weight value overall is greater at 0.8 as it is directly involved in the activation of *P*53.

The hierarchy of interactions in the *P*53 network was utilized to assign vertices to the fuzzy Helm graph, where *P*53 was considered the central vertex and proteins that interacted with it were considered as cycle vertices. Proteins with weaker or secondary interactions are represented as pendant vertices, including *NF*-*kB*, which was assigned to the pendant set to reflect its indirect regulatory role in the *P*53 network. The fuzzy Helm graph was chosen because it captures both central and peripheral interactions. Although other graph structures, such as star or wheel graphs, could also be used, they may produce different structural interpretations by emphasizing only specific interaction patterns.

### Assigning fuzzy weights in protein interaction model

In the fuzzy Helm graph representation of the P53 protein interaction network, membership values for vertices (proteins) and edges (interactions) were assigned based on the confidence levels derived from experimental and computational interaction data. Specifically, we utilized the STRING database, a widely accepted resource that provides interaction confidence scores based on curated experimental results, co-expression, co-occurrence, and text mining. Each edge in the interaction network was associated with a confidence score between 0 and 1, which was directly mapped to the edge membership value. Node membership values were assigned according to the relative biological importance and interaction influence of the corresponding proteins within the P53 regulatory network. Higher membership values were associated with proteins that had stronger regulatory and interaction significance.

The fuzzy weights assigned to protein interactions represent the strength or confidence of the interactions based on biological data, such as binding affinity, expression levels, and experimental validation. These values were obtained from various sources, such as STRING, BioGRID, and UniProtKB. For instance, the relationship between *P*53 and *MDM*2 is well established with a high confidence level; therefore, a fuzzy weight of 0.9 is attributed to this relationship, whereas the interaction associated with *USP*7 through *ATM* contributes indirectly to *P*53 stabilization and is assigned a moderate confidence weight of 0.55. In chemical graph theory, fuzzy topological indices have many applications, as molecular structures are described by graphs with atoms and bonds, and there may be differences in bond strengths in chemical compounds, in the distribution of electrons, or in experimental conditions, leading to uncertainty. Unlike traditional degree-based descriptors, fuzzy topological indices contain membership values that allow for more realistic modeling of real but uncertain molecular interactions. Therefore, these descriptors can be used to investigate molecular stability, predict physicochemical properties, analyze structure–activity relationships, and aid drug discovery and molecular network analysis. The vertex assignments, edge connections, and membership values employed in the P53 fuzzy Helm graph are explicitly shown in Fig. 5 and are consistently used in all subsequent numerical computations, ensuring the full reproducibility of the illustrative example.

### Protein degree computation for the P53 network

To enable full reproducibility of the numerical results in Table 4, we first computed the fuzzy degree of each protein in the P53 network from the edge weights shown in Fig. 5.

**Table 3:** Fuzzy degrees of proteins in the P53 interaction network.

**Vertex**	*μ (v)*	$\deg (v) = \sum$ incident edge weights
P53 (hub)	1.00	*0.9 + 0.85 + 0.88 + 0.9 = 3.53*
MDM2 (cycle)	0.90	*0.9 + 0.75 + 0.82 + 0.6 = 3.07*
ATM (cycle)	0.85	*0.85 + 0.75 + 0.78 + 0.55 = 2.93*
Rb (cycle)	0.88	*0.88 + 0.78 + 0.8 + 0.7 = 3.16*
P21 (cycle)	0.90	*0.9 + 0.8 + 0.82 + 0.8 = 3.32*
SUMO (pendant)	0.60	0.60
USP7 (pendant)	0.55	0.55
NF-*κ*B (pendant)	0.70	0.70
CHK2 (pendant)	0.80	0.80

Using these degrees, we computed all eight fuzzy topological indices. For example, the fuzzy first Zagreb index is

$$\begin{aligned} \mathbb {M}_1(G_f)= & \sum _{p_\alpha \in \alpha _f} \mu _{\alpha _f}(p_\alpha )\cdot (\deg (p_\alpha ))^2 \\= & 1 \cdot (3.53)^2 + 0.9(3.07)^2 + 0.85(2.93)^2 + 0.88(3.16)^2 \\ & + 0.9(3.32)^2 + 0.6(0.6)^2 + 0.55(0.55)^2 + 0.7(0.7)^2 + 0.8(0.8)^2 \\= & 12.4609 + 8.4824 + 7.2922 + 8.7853 + 9.9202 + 0.216 + 0.1664 + 0.343 + 0.512 \\= & 48.1853 \end{aligned}$$

### Numerical results and discussion

In this section, the numerical results of the proposed fuzzy topological indices for the fuzzy Helm graph are presented and analyzed. These results provide useful information regarding the structural properties, connectivity, and uncertainty behavior of the graph. The fuzzy topological indices computed in this study provide useful information regarding the structural and functional behavior of biological networks, particularly the P53 protein interaction pathway. In such networks, proteins are treated as vertices, whereas their interactions are represented by edges. The fuzzy degree associated with each protein reflects the uncertain or partial nature of the biological interactions. Indices such as the fuzzy F-index and fuzzy Zagreb index give greater importance to highly connected vertices and can therefore help identify hub proteins. These proteins are involved in many interactions and often play significant roles in cellular processes. As hub proteins are frequently linked to disease-related mechanisms, they are considered important candidates for drug target research.

The fuzzy Randic and Harmonic indices provide additional insights into the distribution and strength of connections within the network. In many cases, a smaller fuzzy Randic index indicates a more centralized or highly branched structure, which may reflect the existence of an influential or highly interactive protein. Furthermore, the fuzzy Misbalance Prodeg and fuzzy Sombor indices help identify structural irregularities and asymmetrical interaction patterns in the network. Such variations may indicate important regulatory regions or bottleneck points involved in the signal transduction processes. The fuzzy Nirmala index, which considers the combined contribution of interacting node pairs, can also assist in detecting not only hub proteins but also significant interaction pathways that may be valuable for therapeutic and drug target investigations.

In the proposed modeling of the P53 pathway, proteins associated with larger fuzzy F-index and fuzzy Zagreb index values correspond to highly connected vertices in the illustrative P53 network, suggesting their potential importance within the modeled interaction framework. They are known as “markers” in cancer research and thus indicate the possible relevance and applicability of fuzzy topological analysis in the case of proteins with high functional relevance for uncertain or imprecise network data. To analyze this fuzzy protein network, we computed several fuzzy topological indices. The computed values and their interpretations are given below See Table 4.

**Table 4:** Summary of Computed Fuzzy Topological Indices for the P53 Network.

*Index*	Computed Value	Biological Interpretation
Fuzzy First Zagreb Index	48.18533	High value suggests that P53 is a central regulatory hub, controlling multiple pathways.
Fuzzy Second Zagreb Index	37.15170	Strong connectivity in the pathway suggests high functional importance of direct interactions.
Fuzzy Randic Index	3.23754	Lower value suggests possible alternative drug targets, such as *SUMO* or *USP*7 for pathway modulation.
Fuzzy Harmonic Index	1.31189	Illustrates that secondary regulators (pendant nodes) have weaker contributions but still influence the network.
Fuzzy F-index	129.16267	High value emphasizes the dominant regulatory role of highly connected proteins like P53.
Fuzzy Misbalance Prodeg Index	36.711	Indicates structural irregularities in the interaction network, suggesting regulatory bottlenecks.
Fuzzy Sombor Index	44.70217	A high value suggests that P53 and its primary interactors (*MDM*2, *ATM*, *Rb*, *P*21) play dominant roles in cancer progression.
Fuzzy Nirmala Index	26.567	Reflects the combined connectivity strength, highlighting significant interaction pathways.

### Comparison with classical degree-based topological indices

The numerical behavior of the proposed fuzzy topological indices is closely related to that of classical degree-based indices. However, unlike classical descriptors, fuzzy indices incorporate membership values associated with vertices and edges, allowing uncertain and partial relationships to be modeled more effectively. Moreover, when all membership values are equal to one, the proposed fuzzy indices reduce to their corresponding classical degree-based forms. Thus, fuzzy topological descriptors can be viewed as generalized extensions of classical indices for analyzing uncertain graph structures.

### Comparison with existing models

Traditional crisp Helm graphs model perfect relationships between nodes, assuming the binary (0 or 1) presence of vertices and edges. While useful for structural analysis, such models cannot capture the inherent uncertainty and gradation of real biological interactions in the human body. Unlike the fuzzy Helm graph, the fuzzy graph approach can represent fuzzy memberships, which is more realistic for modeling the varying strengths, confidence, and experimental evidence of protein interactions. Compared to other fuzzy graph models applied in bioinformatics, such as fuzzy path graphs, molecular graphs, and fuzzy flower and star graphs, our approach specifically captures both centralized hub behavior (via the central vertex) and peripheral uncertainty (via pendant vertices). This mixed structural behavior is very similar to that of real biological protein networks, in which certain proteins in the “hub” (P53) are bound to a partner protein that is either stable or temporary. The fuzzy molecular graph has been successfully used to represent the atomic structure, while the fuzzy flower or star graph has been successfully used to represent the radial connectivity pattern; however, none of these models can represent the layered symmetry and connectivity pattern found in the Helm graph. The proposed fuzzy Helm graph is thus a more flexible and informative view of biological systems, such as the (P53) pathway, which incorporates central dominance and varying degrees of interaction strengths. Moreover, with the use of degree-based fuzzy indices, a detailed examination of the importance of nodes, structural irregularity, and biological relevance of the graph can be accomplished, which is not possible with simpler graph models, providing a higher degree of both structural and biological interpretability, making it a promising alternative to classical crisp and some existing fuzzy graph models.

### Chemical graph representation of a molecular structure

Chemical graph theory has wide applications in the study of molecular structures using graph models. To demonstrate the usefulness of the proposed fuzzy Helm graph framework in chemistry, we considered a simplified molecular structure derived from aromatic hydrocarbons. In this model, the functional groups are depicted as pendant nodes, and the central atom as the hub vertex, the structure of which is represented by a fuzzy Helm-type graph. Membership values are assigned based on the bond strength and reliability of the molecular interactions.

**Fig. 6:**
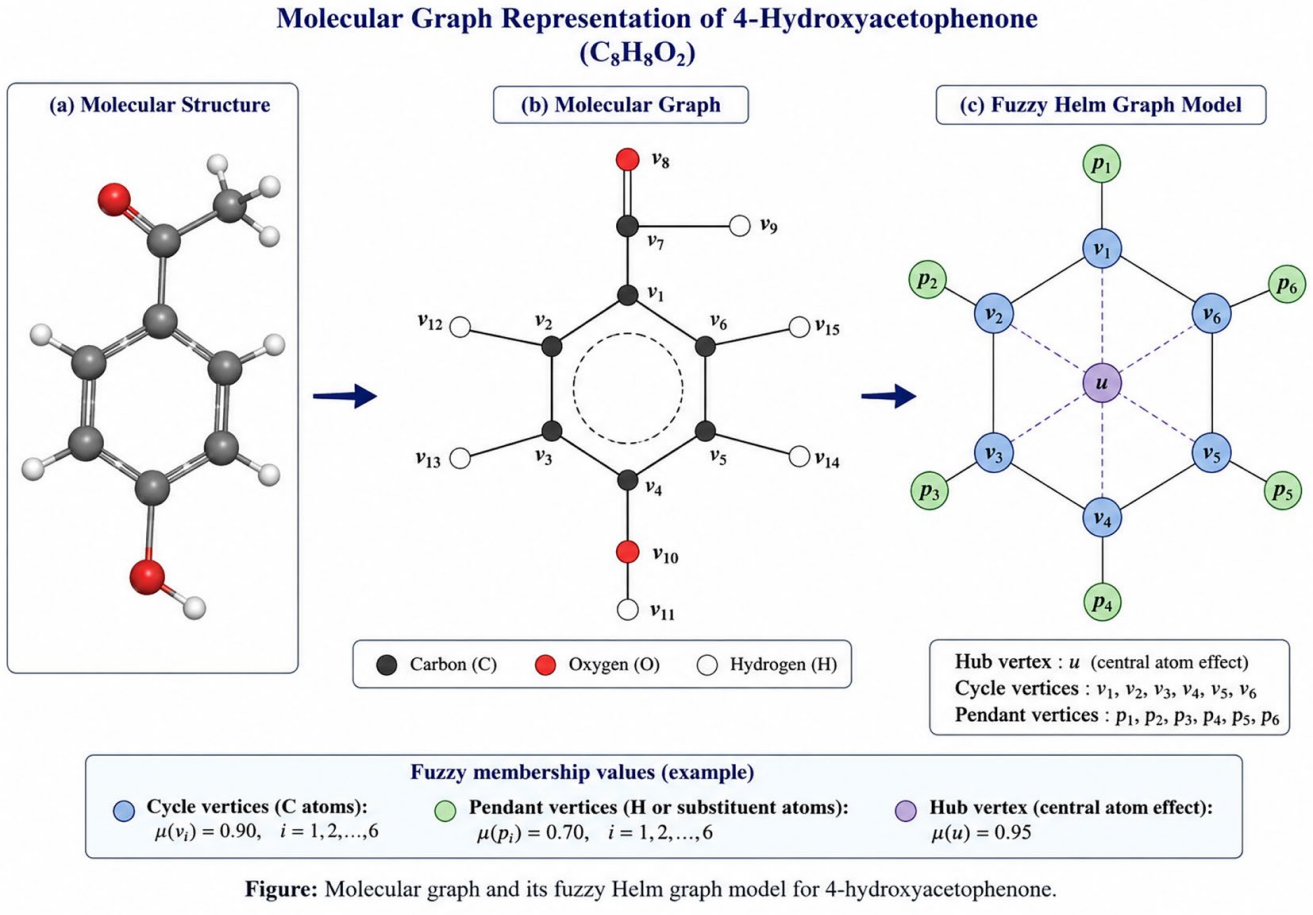
A fuzzy molecular graph representation of a chemical compound using a Helm-type structure.

The molecular structure can be graphically represented as shown in Fig. 6. This example demonstrates that fuzzy topological indices can effectively characterize the molecular connectivity and structural uncertainty of chemical compounds.

## Conclusion

In this study, we present a detailed analysis of several fuzzy topological descriptors for fuzzy Helm graphs. The obtained results demonstrate how these indices reflect different structural characteristics of uncertain graph models and provide useful insights into connectivity behavior under fuzziness. To illustrate the potential application of the proposed framework to uncertain biological networks, an illustrative fuzzy Helm graph representation of an interaction motif for a *P*53 was also used. The proposed methodology can be generalized to other fuzzy graph families, such as temporal fuzzy graphs, multilayer fuzzy networks, and generalized uncertain interaction systems (GIS). In general, the present study is helpful as a contribution to the body of research on the topologically fuzzy approach to structured graph families. The biological example provided is not an exhaustive representation of a biological network model but rather a representative example of the proposed fuzzy Helm graph model. This study presents a computational model that enables the analysis of structured graph families with uncertainty using fuzzy topological descriptors. The derived formulations and methodology may be expanded to other classes of fuzzy graphs that are related and can be used to facilitate further research on uncertain models of chemical, biological, and communication networks.

## Limitations and future directions

This study is limited to fuzzy Helm graphs, and the results obtained may not be directly applicable to other fuzzy graph classes. The computational complexity of analyzing these fuzzy topological indices, specifically for large Helm graphs with fuzziness, remains unexplored. Future studies could extend this analysis to larger protein interaction networks to study neurodegenerative diseases or metabolic disorders. Additionally, machine learning techniques integrated with fuzzy Helm graphs can be used to predict drug interactions more accurately. It would also be valuable to compute and illustrate these topological indices using real-world biological data from protein databases, such as STRING or BioGRID.

## Data Availability

The datasets used and/or analysed during the current study available from the corresponding author on reasonable request.
